# Immunological and inflammatory mapping of vascularized composite allograft rejection processes in a rat model

**DOI:** 10.1371/journal.pone.0181507

**Published:** 2017-07-26

**Authors:** Or Friedman, Narin Carmel, Meirav Sela, Ameen Abu Jabal, Amir Inbal, Moshe Ben Hamou, Yakov Krelin, Eyal Gur, Nir Shani

**Affiliations:** The Plastic Reconstructive Surgery Department, Tel Aviv Sourasky Medical Center, Sackler Faculty of Medicine, Tel Aviv University, Tel Aviv, Israel; University of Kentucky, UNITED STATES

## Abstract

**Background:**

Hand and face vascularized composite allotransplantation (VCA) is an evolving and challenging field with great opportunities. During VCA, massive surgical damage is inflicted on both donor and recipient tissues, which may contribute to the high VCA rejection rates. To segregate between the damage-induced and rejection phase of post-VCA responses, we compared responses occurring up to 5 days following syngeneic versus allogeneic vascularized groin flap transplantations, culminating in transplant acceptance or rejection, respectively.

**Methods:**

The immune response elicited upon transplantation of a syngeneic versus allogeneic vascularized groin flap was compared at Post-operative days 2 or 5 by histology, immunohistochemistry and by broad-scope gene and protein analyses using quantitative real-time PCR and Multiplex respectively.

**Results:**

Immune cell infiltration began at the donor-recipient interface and paralleled expression of a large group of wound healing-associated genes in both allografts and syngrafts. By day 5 post-transplantation, cell infiltration spread over the entire allograft but remained confined to the wound site in the syngraft. This shift correlated with upregulation of IL-18, INFg, CXCL9, 10 and 11, CCL2, CCL5, CX3CL1 and IL-10 in the allograft only, suggesting their role in the induction of the anti-alloantigen adaptive immune response.

**Conclusions:**

High resemblance between the cues governing VCA and solid organ rejection was observed. Despite this high resemblance we describe also, for the first time, a damage induced inflammatory component in VCA rejection as immune cell infiltration into the graft initiated at the surgical damage site spreading to the entire allograft only at late stage rejection. We speculate that the highly inflammatory setting created by the unique surgical damage during VCA may enhance acute allograft rejection.

## Introduction

Vascularized composite allotransplantation (VCA) is the single-piece transfer of a composite tissue that may include skin, muscle, bone, blood vessels and nerves. It has the potential to revolutionize the field of reconstructive surgery, by providing a perfect "replacement part” for tissues compromised by disease or trauma. It is the only procedure, thus far, that bears the potential to restore near-normal appearance in patients with socially crippling facial injuries, and offers the most complete functional restoration currently available for hand amputees. To date, over 100 hand transplantations and 37 face transplantations have been successfully performed worldwide [1–9]. However, similarly to other foreign grafts, VCA grafts are rejected by the recipient’s immune system unless a strict immunosuppressive regimen is given to the recipients throughout their life, often leading to severe side effects [10, 11]. Naturally, both face and hand transplantations inflict far more surgical damage to both the recipient and donor tissue as compared to transplantation of internal organs resulting in larger surface area of disrupted and damaged tissue. This may partly explain the rate of acute graft rejections within the first year of such transplantations, which is 85% in hand transplantations and 84% in face transplantations, higher than any other field of transplantation [12–15].

Injury-induced inflammation is an ordered process that includes the migration of platelets, neutrophils, macrophages, and lymphocytes into the wound area [16, 17], and is thought to occur through release of damage-associated molecular patterns (DAMPs) of endogenous molecules from injured tissue. The Toll-like receptors (TLRs), receptor for advanced glycation end products (RAGE) and nucleotide-binding oligomerization domain-like receptors (NLRs) [18] have been implicated in this process. Ischemia-reperfusion injury (IRI), prevailing in all allografts, activates TLRs, which, in turn, have been suggested to play a significant role in shifting the balance from healing and tolerance to allograft rejection and in determining the intensity of graft rejection [19–21]. Kidney transplant damage often leads to delayed graft function (DGF), defined as the need for dialysis within one week of the transplantation, and is most prevalent amongst patients receiving cadaveric kidneys characterized by an advanced inflammatory state [22]. Interestingly, a strong correlation was observed between the occurrence of DGF and kidney transplant rejection [23]. Thus, although allograft rejection is ultimately dictated by the adaptive immune response against foreign donor antigens, the intensity of the rejection response is highly affected by the initial inflammatory response that is controlled by the degree of the recipient and donor tissue damage [24–26].

The aim of the current study was to distinguish between the cues that drive damage-induced inflammation versus the adaptive immune response against foreign donor antigens during VCA rejection. To this end, the immune response elicited upon transplantation of a syngeneic versus allogeneic vascularized groin flap was compared by histology and by broad-scope gene and protein analyses. The vascularized groin flap used in the study is the most basic VCA model comprised of skin, blood vessels, nerve, fat and muscle. This vascularized graft partly resembles a face transplant, since it is composed mainly of skin and does not contain a dominant bone fraction, which introduces a substantial donor hematopoietic component. It was assumed that syngeneic transplantation induces an acute damage-triggered inflammatory response only, while allogeneic transplantation evokes both an acute inflammatory response and an antigen-dependent adaptive immune response. The surgical procedure required for this model introduces extensive surgical damage to both the recipient flap bed and to the donor flap perimeter, providing an adequate model for studying the possible contribution of an injury-associated acute inflammatory component to VCA rejection.

## Materials and methods

### Animals

Inbred Lewis rats (LEW; RT1^**l**^ strain) and Brown Norway (BN) rats (RT1^N^), aged 12 weeks and weighing 280–320g, were used in the study. Animals were maintained under standard, controlled conditions. The study was conducted in accordance with the Guide for the Care and Use of Laboratory Animals and approved by the Institutional Animal Care and Use Committee of Tel Aviv Sourasky Medical Center.

### Animal vascularized groin flap model

The well-described groin free-flap rat model was used [27]. This composite flap, composed of skin, panniculus carnosum and subcutaneous fat, was raised above the abdominal wall. Irrigation with 10 ml Ringer Lactate with 50 U heparin was performed via the flap’s femoral artery, until clear fluid could be seen exiting the femoral vein. The donor vessels were anastomosed end-to-end with the host femoral vessels, using a 10–0 nylon interrupted suture. Syngeneic groin free-flap transfers in performed on recipient Lewis rats from transplanted with grafts from donor Lewis rats and allogeneic groin free-flap transfers were performed recipient Lewis rats transplanted with grafts from donor BN rats.

### Experimental design

Rats underwent either a syngeneic or allogeneic groin free flap transfer and were sacrificed on either post-operative day (POD) 2 or POD 5. Tissue samples were collected from both the donor-recipient interface and from the middle of the graft, for histological, immunohistochemical, qRT-PCR and protein analyses. Normal undamaged skin from the same rat served as the control for all tests. The animals used in the experiments described in the study are detailed in Table 1. Additional ~40 allogeneic transplantations were performed during the calibration of the transplantation procedure and selecting the exact time points after transplantation that are presented in the study.

**Table 1:** Animals used in the study.

	Syngeneic transplantation	Allogeneic transplantation
POD 2	5 animals	5 animals
POD 5	5 animals	3 animals
Total	10 animals	8 animals

### Sample preparation

Biopsies were sliced into three sections and stored in either liquid nitrogen, for RNA and protein analyses, or in 4% formaldehyde for histological analysis.

### RNA isolation and quantitative real-time PCR (qRT-PCR)

Tissues were defrosted and homogenized using a Tissue homogenizer and then digested with proteinase K. Total RNA was isolated using the gen Elute Mammalian Total RNA Miniprep Kit (Sigma-Aldrich, Israel). Total RNA was reverse-transcribed using the Reverse Transcription Kit (Quanta Bioscience) and qRT-PCR was performed with a Real-Time PCR System (Applied Biosystems), using SYBR green (Quanta Bioscience). Expression levels were normalized to HPRT1 or Rn18s. Primer sequences are presented in S1 Table. C_T_ values were determined by automated threshold analysis. Fold change for each gene was calculated using the ΔΔC_t_ method. Each sample was tested in triplicate.

### Lysate preparation

Tissue samples (400mg) were homogenized in Tris buffer solution (Tris base 7.6pH (50mM), NaCl 150mM, EDTA 5Mm and protease inhibitor cocktail (1:100) (Sigma-Aldrich, Israel), with a Tissue homogenizer and centrifuged at 14,000 rpm (4°C, 10min) and stored in -80°C. Protein concentration was measured by bicinchoninic acid (BCA) assay (Pierce, USA). Lysate samples were evaluated by Multiplexing LASER Bead Technology (Eve Technologies, Calgary, Canada).

### Multiplex analysis of cytokines

In this study we quantified 27 cytokine/ chemokine biomarkers simultaneously by using a Discovery Assay^®^ called the Rat Cytokine Array/ Chemokine Array 27-Plex (Eve Technologies Corp, Calgary, AB, Canada). The multiplex assay was performed at Eve Technologies by using the Bio-Plex^™^ 200 system (Bio-Rad Laboratories, Inc., Hercules, CA, USA), and a Milliplex rat cytokine kit (Millipore, St. Charles, MO, USA) according to their protocol. The 27-Plex consisted of EGF, Eotaxin, Fractalkine, G-CSF, GMCSF, GRO/KC/CINC-1, IFNγ, IL-1α, IL-1β, IL-2, IL-4, IL-5, IL-6, IL-10, IL-12(p70), IL-13, IL-17A, IL-18, IP-10, Leptin, LIX, MCP-1, MIP-1α, MIP-2, RANTES, TNFα and VEGF. The assay sensitivities of these markers range from 0.1–15.7 pg/mL. Individual analyte values and other assay details are available on Eve Technologies' website or in the Milliplex protocol.

### Histology

Sections were either stained only by Hematoxylin and Eosin (H&E) or processed by immunohistechemical staining. Immunohistochemical analysis was performed on 4 *μ*m-thick paraffin-embedded sections. Sections were deparaffinised and rehydrated. Antigen retrieval was performed (0.01 M citrate, pH 6.0) and sections were rinsed in phosphate-buffered saline (PBS). Single-labelling immunohistochemistry was performed according to the manufacturer’s instructions of the mouse and rabbit specific HRP/AEC (ABC) detection IHC kit (Abcam, Cambridge, UK). Briefly, sections were stained with primary antibodies: mouse anti rat CD68 (AbD serotec, USA), rabbit anti rat CD4 (Novus Biologicats, Canada) and mouse anti rat CD8a (BD Pharmingen), washed and incubated with HRP-goat anti-mouse/rat/rabbit IgG, visualized with substrate-chromagen AEC, counterstained with haematoxylin and mounted with Immuno-mount (Thermo USA).

### Statistical analysis

Data are expressed as mean ± standard deviation of independent experiments, each done at least in triplicate. Statistical evaluation of the data was done using Student’s t-test (SPSS 23 software for Windows). P values < 0.05 (*) were considered statistically significant.

## Results

### Allogeneic immune infiltration is initiated at the host-donor interface proximal to the site of surgical damage

**Fig 1:**
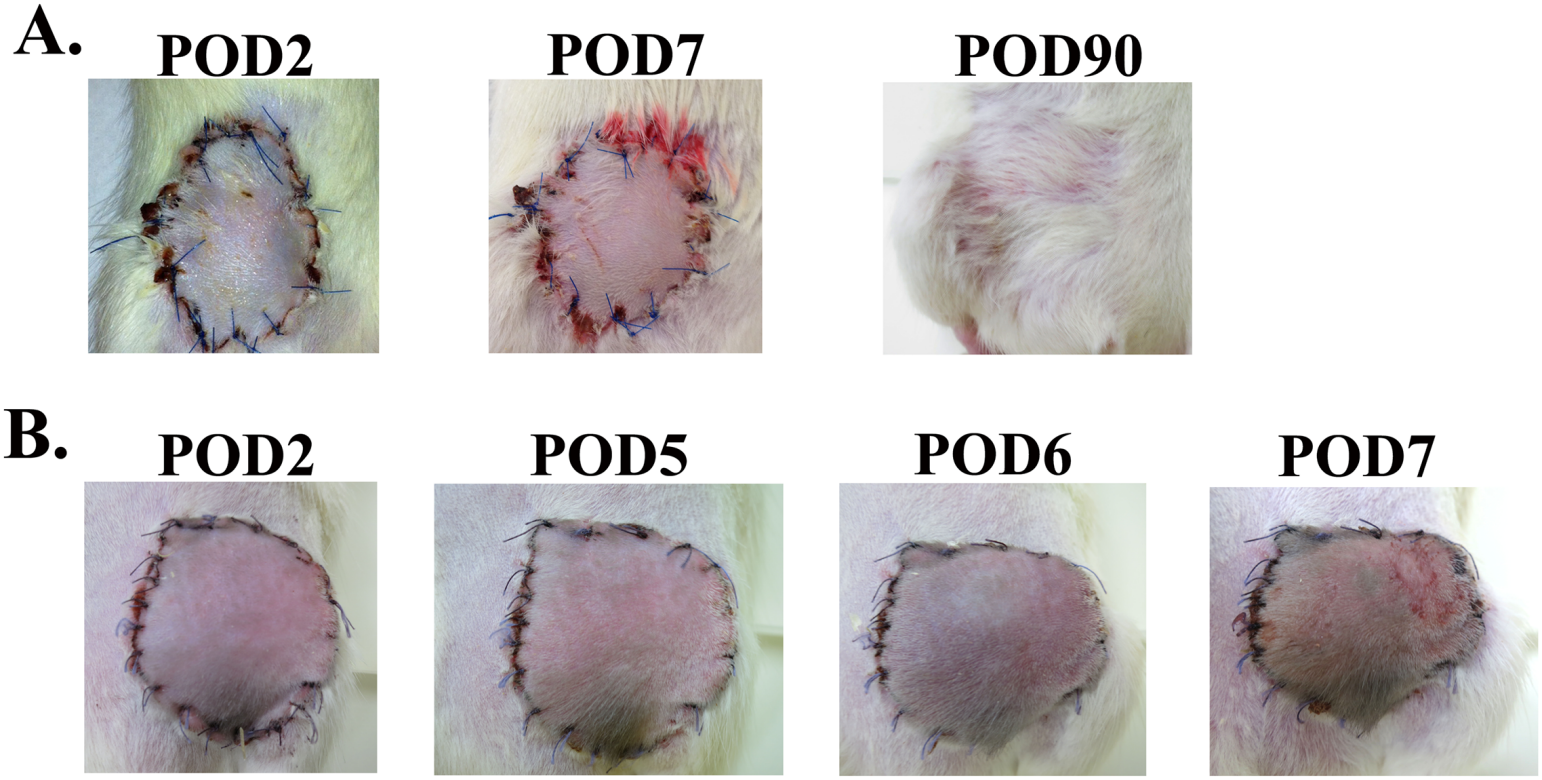
Acute allograft rejection is clinically manifested in the vascularized groin flap model between POD 5 and 6. Monitoring of the groin flaps post op. (A) Syngraft at POD 2, 7 and 90. Note the regular non edematous surface good perfusion with no discoloration at POD 2 and 7 and complete healing with only the different direction of hair growth to indicate the syngraft location. (B) Allograft at POD 2, 5, 6, 7. Note that the POD 2 allograft already shows early signs of inflammation, edema, red color indicating hyperemia. POD 5 and 6 allograft shows clinical signs of rejection, patches of different colors indicating necrosis and extremely swollen and hard on palpation. POD 7 allograft is completely rejected and non-viable.

**Fig 2:**
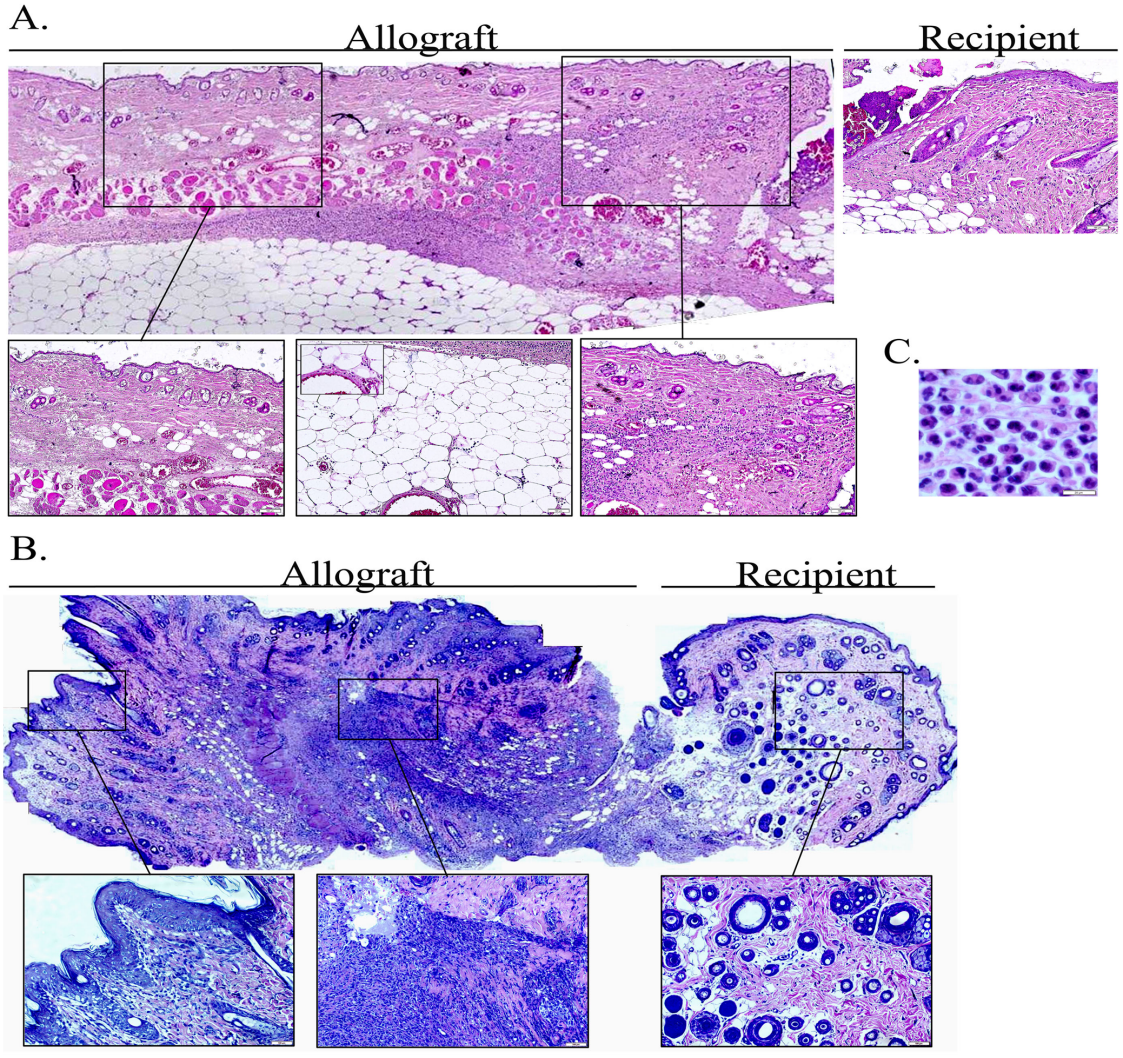
Allograft immune rejection initiates at the donor-recipient interface and then spreads to the entire allograft. Samples from the allograft-recipient interface were removed on POD 2 (A) or POD 5 (B) and paraffin sections were stained with hematoxylin-eosin (H&E). A panoramic view made by stitching images, is presented. Insets of the original images are displayed to provide a higher image resolution of selected regions. (C) An enlarged view of the inflamed zone demonstrates the high granulocyte content amongst infiltrating leukocytes.

**Fig 3:**
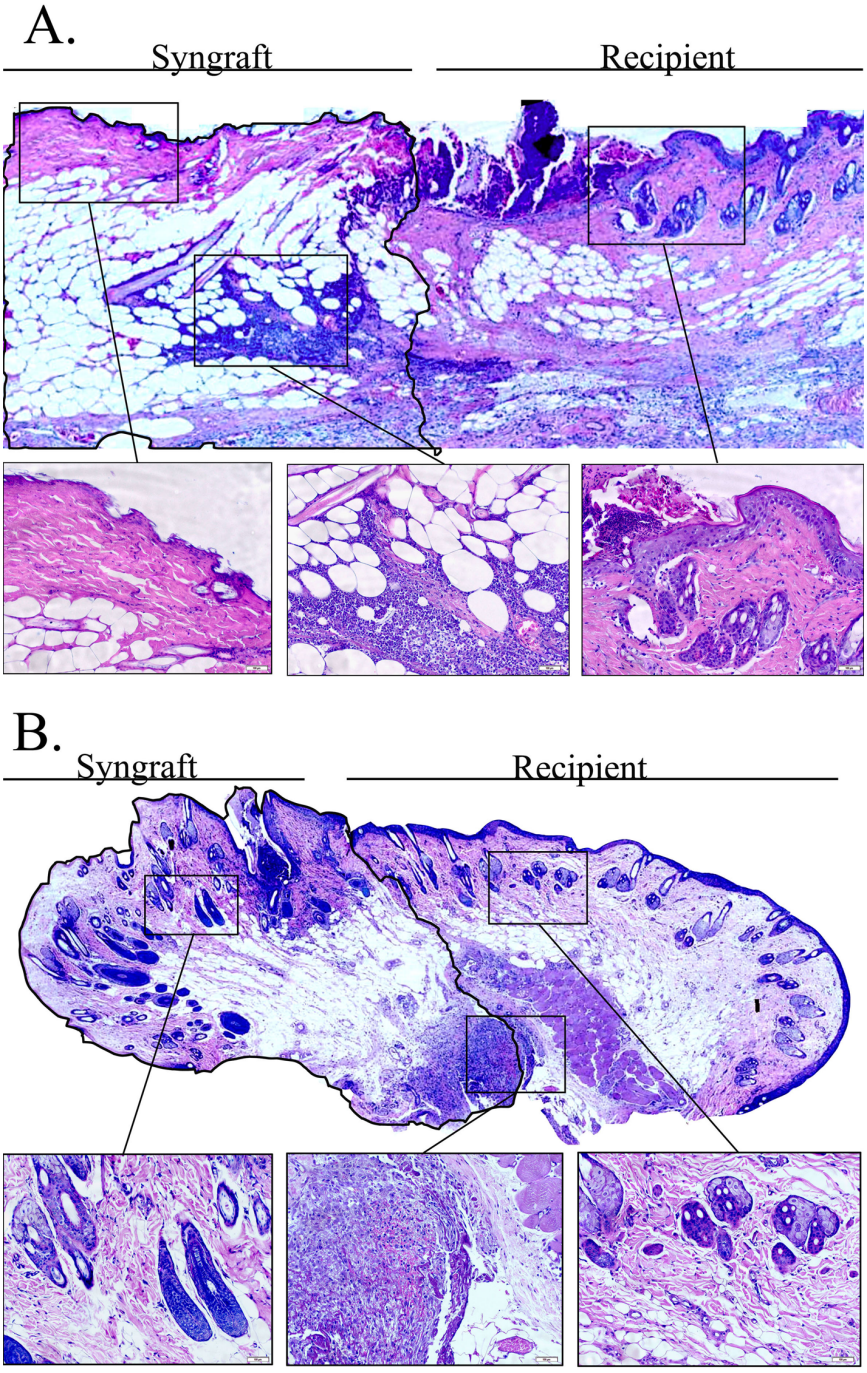
Inflammation in syngrafts remains concentrated at the graft-recipient interface through POD 5. Samples from the syngraft-recipient interface were removed on POD 2 (A) or POD 5(B) and paraffin sections were stained with H&E. A panoramic view made by stitching images, is presented. Insets of the original images are displayed to provide a higher image resolution of selected regions. The area of the syngraft is surrounded by a black line.

**Fig 4:**
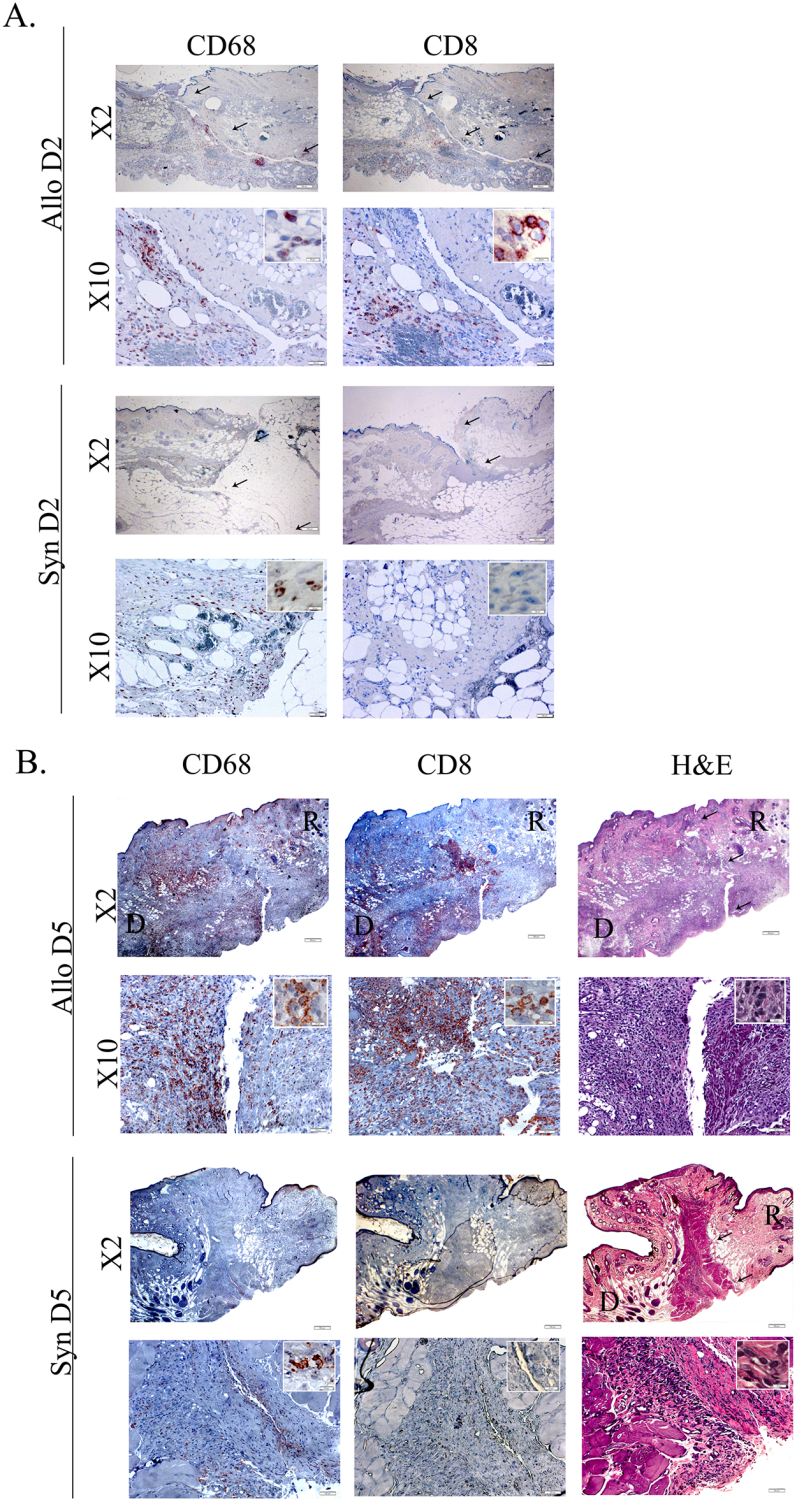
CD8^+^ and CD68^+^ cell infiltration initiates at the donor-recipient interface at POD 2 and then spreads to the entire allograft. Samples from the graft-recipient interface of allografts and syngrafts were removed on POD 2 (A) and POD 5 (B). Paraffin sections were stained with anti-CD68 or anti-CD8 antibodies, followed by hematoxylin counter staining (A and B) or stained only by H&E (B). Arrows mark the donor recipient interface. Donor (D), recipient (R).

**Fig 5:**
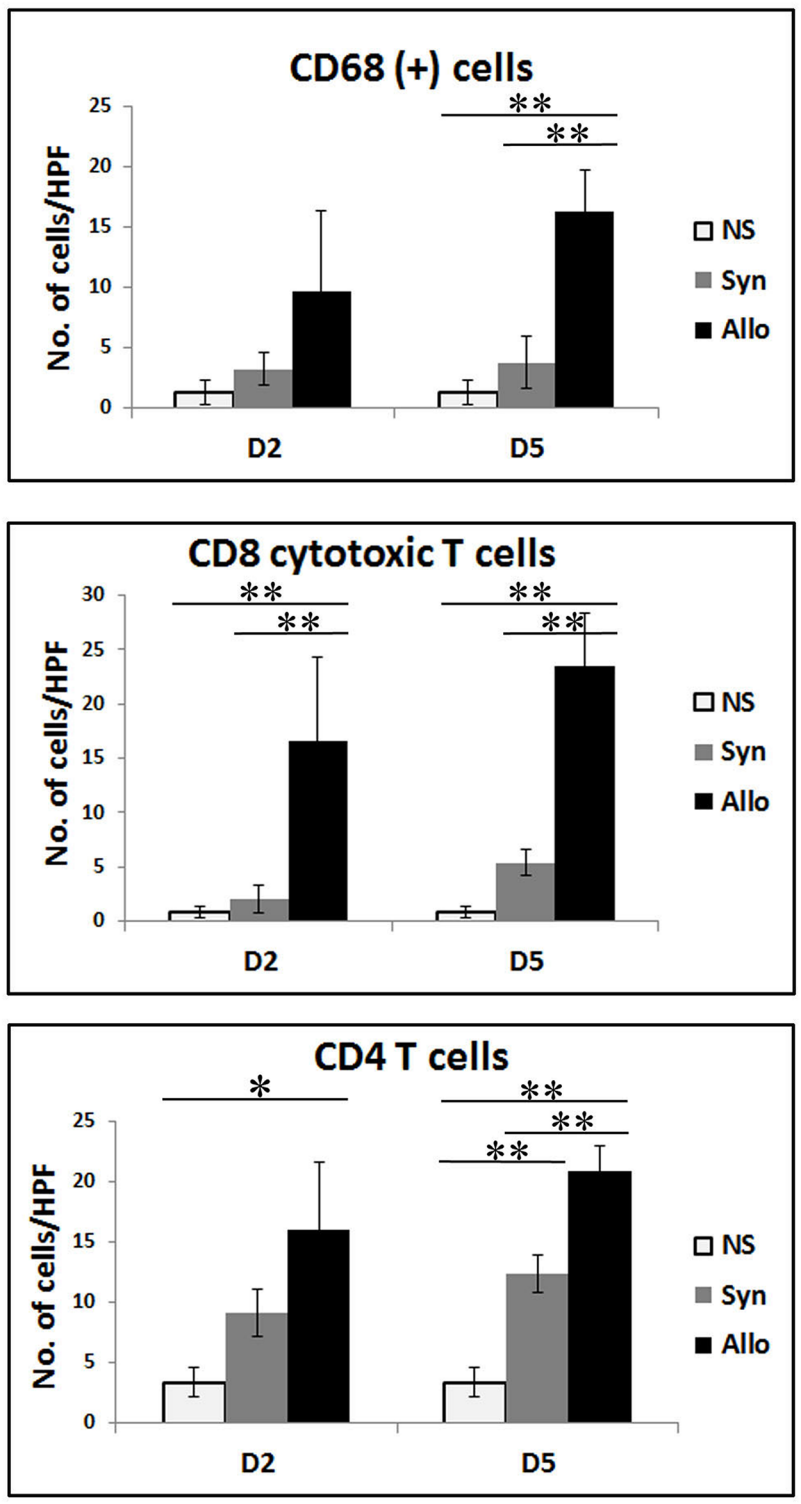
Immunohistochemical quantification of cell infiltration into allografts, syngrafts and normal skin (NS). Samples (n = 3–5 rats per group) from the graft-recipient interface of a syngraft, allograft or from NS were removed at the indicated times and paraffin sections were stained with anti CD68, CD8 or CD4 antibodies followed by hematoxylin counterstaining. Stained cells were counted in 10 fields and the average stained cells per field is displayed in the graphs. * P<0.05 and ** P<0.01.

AS can be seen in Fig 1A and 1B examination of syngeneic and allogeneic grafts at the days following transplantation revealed no significant clinical signs of inflammation up to POD 2 in both graft types. Signs of acute rejection were first observed in the allograft between POD 5 and 6 when it became swollen, hard and red within a few hours (Fig 1B). The rejection process was swift and allografts became hard and pale at POD 7 (Fig 1B), most likely due to blood flow obstruction marking complete rejection. As can be seen at Fig 1A unlike the allograft, the syngraft remained clinically unaffected in the first 7 days post transplantation followed by its full acceptance. Thus, in order to compare the temporal advance of the immune responses elicited by syngeneic and allogeneic transplantation, grafts were analyzed on POD 2 and POD 5, which represent the inflammatory (damage response) and acute rejection phases, respectively. Examination of H&E-stained POD 2 histological sections revealed an acute inflammatory response in both allogeneic and syngeneic grafts, which was only visible at the graft-recipient interface, but not in other graft or host regions (Figs 2A and 3A, respectively), including the center of POD 2 allografts (S1 Fig). Many of the inflammatory cells infiltrating the allograft skin were found in a perivascular position within the subcutaneous fat (Fig 2A) and appeared to migrate towards the damaged area in the skin. The inflamed region in both graft types contained mostly granulocytes (Fig 2C), and mild infiltration of CD68^+^ cells (macrophages) (Fig 4A). Infiltrating CD4^+^ cells (T helper cells) were also detected (Fig 5). Pronounced infiltration of CD8^+^ cells (cytotoxic lymphocytes) was observed in the allogeneic grafts only (Fig 4A). On POD 5, immune cell infiltration in the syngeneic graft remained restricted to the vicinity of the graft-recipient border, while in the allogeneic graft, infiltration spread linearly from the graft-recipient border to the outer allograft perimeter (Figs 2B versus 3B). Infiltrating cells included CD68^+^ and CD4^+^ cells in both graft types. Pronounced infiltration of CD8^+^ cells, however, was observed in the allogeneic grafts only (Figs 4B and 5). As anticipated, granulocytes were not detected in either the syngeneic or allogeneic grafts at this stage (data not shown).

### Distinct gene expression profiles of damage-related inflammatory responses versus adaptive anti-alloantigen immune responses

To better characterize the molecular events that control the post-transplantation inflammatory response, RNA expression patterns of various immune modulators in allografts and syngrafts were compared to those of normal (undamaged) skin (NS), using qRT-PCR. Expression of IL-1b, IL-6, IL-10, IFNg, TGFb, CCL2, CCL3, CCL4, CCL5, CXCL1 and CXCL2 (group 1) was significantly increased by POD 5 in both syngeneic and allogeneic grafts compared to NS (Table 2), indicating their involvement in damage-related inflammation. In contrast, IL-18, TNFa, CCL7, CCL17, CX3CL1, CXCL9, CXCL10 and CXCL11 (group 2) were significantly upregulated in the allogeneic grafts only (Table 2), indicating their involvement in the adaptive anti-alloantigen immune response. Expression patterns of IL-2, IL-17, IL-23, IL-4, IL-5, GM-CSF, CCL20 and CCL22 (group 3) were unchanged compared to NS, in both syngeneic and allogeneic grafts (Table 2), indicating that they are unrelated to both damage responses and VCA rejection in this model within the examined time period. A significant increase in CCL21 expression was observed in the syngrafts only (Table 2). Importantly, many of these trends were already detected on POD 2, but were still insignificant at that stage (S2 Table).

**Table 2:** Comparison of the RNA expression of an array of immune modulators of syngeneic or allogeneic grafts 5 days post transplantation with normal (uninflamed) skin (NS).

		Average NS	Average Syn D5	Fold Av Syn D5/Av NS	P		Average NS	Average *Allo* D5	Fold Av Allo D5/Av NS	P
1. Syn & Allo	**IL-1a**	**3395.48**	**6046.26**	**1.78**	**0.141**	**IL-1a**	**3395.48**	**10019.48**	**2.95**	**0.016**
	**IL-1b**	**3220.67**	**29366.44**	**9.12**	**0.010**	**IL-1b**	**3220.67**	**45074.33**	**14.00**	**0.003**
	**IL-6**	**197.84**	**2488.44**	**12.58**	**0.002**	**IL-6**	**197.84**	**7584.79**	**38.34**	**0.001**
	**IL-10**	**17.94**	**62.59**	**3.49**	**0.015**	**IL-10**	**17.94**	**557.45**	**31.07**	**0.001**
	**IL-12a**	**375.13**	**119.09**	**0.32**	**0.004**	**Il-12a**	**375.13**	**126.94**	**0.34**	**0.005**
	**IFNy**	**188.84**	**1041.12**	**5.51**	**0.001**	**IFNy**	**188.84**	**136123.14**	**720.83**	**0.002**
	**TGFb**	**5188.24**	**11423.00**	**2.20**	**0.031**	**TGFb**	**5188.24**	**13265.35**	**2.56**	**0.039**
	**CCL2**	**5187.19**	**42666.07**	**8.23**	**0.001**	**CCL2**	**5187.19**	**288356.66**	**55.59**	**0.002**
	**CCL3**	**0.08**	**1.71**	**20.61**	**0.001**	**CCL3**	**0.08**	**2.30**	**27.73**	**0.015**
	**CCL4**	**404.64**	**3711.73**	**9.17**	**0.000**	**CCL4**	**404.64**	**17811.48**	**44.02**	**0.022**
	**CCL5**	**1842.43**	**4516.61**	**2.45**	**0.019**	**CCL5**	**1842.43**	**15106.48**	**8.20**	**0.000**
	**CXCL1**	**426.28**	**4214.81**	**9.89**	**0.000**	**CXCL1**	**426.28**	**9741.54**	**22.85**	**0.000**
	**CXCL2**	**8419.71**	**601218.24**	**71.41**	**0.004**	**CXCL2**	**8419.71**	**533491.39**	**63.36**	**0.024**
2. Allo only	**CCL7**	**1.03**	**3.34**	**3.23**	**0.109**	**CCL7**	**1.03**	**20.38**	**19.75**	**0.001**
	**IL-18**	**6951.90**	**9354.64**	**1.35**	**0.527**	**IL-18**	**6951.90**	**24199.80**	**3.48**	**0.011**
	**TNFa**	**1126.60**	**949.26**	**0.84**	**0.702**	**TNFa**	**1126.60**	**2572.43**	**2.28**	**0.014**
	**CCL17**	**1290.01**	**1972.25**	**1.53**	**0.375**	**CCL17**	**1290.01**	**3815.52**	**2.96**	**0.040**
	**CX3CL1**	**717.31**	**719.25**	**1.00**	**0.993**	**CX3CL1**	**717.31**	**3531.43**	**4.92**	**0.000**
	**CXCL9**	**4.16**	**7.65**	**1.84**	**0.276**	**CXCL9**	**4.16**	**241.00**	**57.97**	**0.001**
	**CXCL10**	**0.81**	**0.82**	**1.02**	**0.942**	**CXCL10**	**0.81**	**15.72**	**19.44**	**0.000**
	**CXCL11**	**0.01**	**0.10**	**9.62**	**0.248**	**CXCL11**	**0.01**	**9.09**	**888.13**	**0.001**
3. Not involved	**CCL19**	**9177.97**	**30921.76**	**3.37**	**0.052**	**CCL19**	**9177.97**	**109339.73**	**11.91**	**0.060**
	**IL-2**	**119.86**	**68.92**	**0.58**	**0.362**	**IL-2**	**119.86**	**119.68**	**1.00**	**0.997**
	**IL-4**	**8213.45**	**5589.27**	**0.68**	**0.228**	**IL-4**	**8213.45**	**13315.12**	**1.62**	**0.346**
	**IL-5**	**750.94**	**1217.74**	**1.62**	**0.148**	**IL-5**	**750.94**	**922.28**	**1.23**	**0.640**
	**IL-17**	**3460.22**	**3080.95**	**0.89**	**0.819**	**IL-17**	**3460.22**	**5142.84**	**1.49**	**0.414**
	**IL-23**	**1000.43**	**829.01**	**0.83**	**0.615**	**IL-23**	**1000.43**	**1418.04**	**1.42**	**0.459**
	**GM-CSF**	**1194.25**	**900.50**	**0.75**	**0.638**	**GM-CSF**	**1194.25**	**1779.69**	**1.49**	**0.366**
	**CCL20**	**6314.21**	**4522.72**	**0.72**	**0.719**	**CCL20**	**6314.21**	**14459.86**	**2.29**	**0.173**
	**CCL22**	**7567.91**	**5338.33**	**0.71**	**0.539**	**CCL22**	**7567.91**	**10123.27**	**1.34**	**0.454**
4. Syn Only	**CCL21**	**50131.12**	**186690.14**	**3.72**	**0.008**	**CCL21**	**50131.12**	**83108.16**	**1.66**	**0.393**

**Fig 6:**
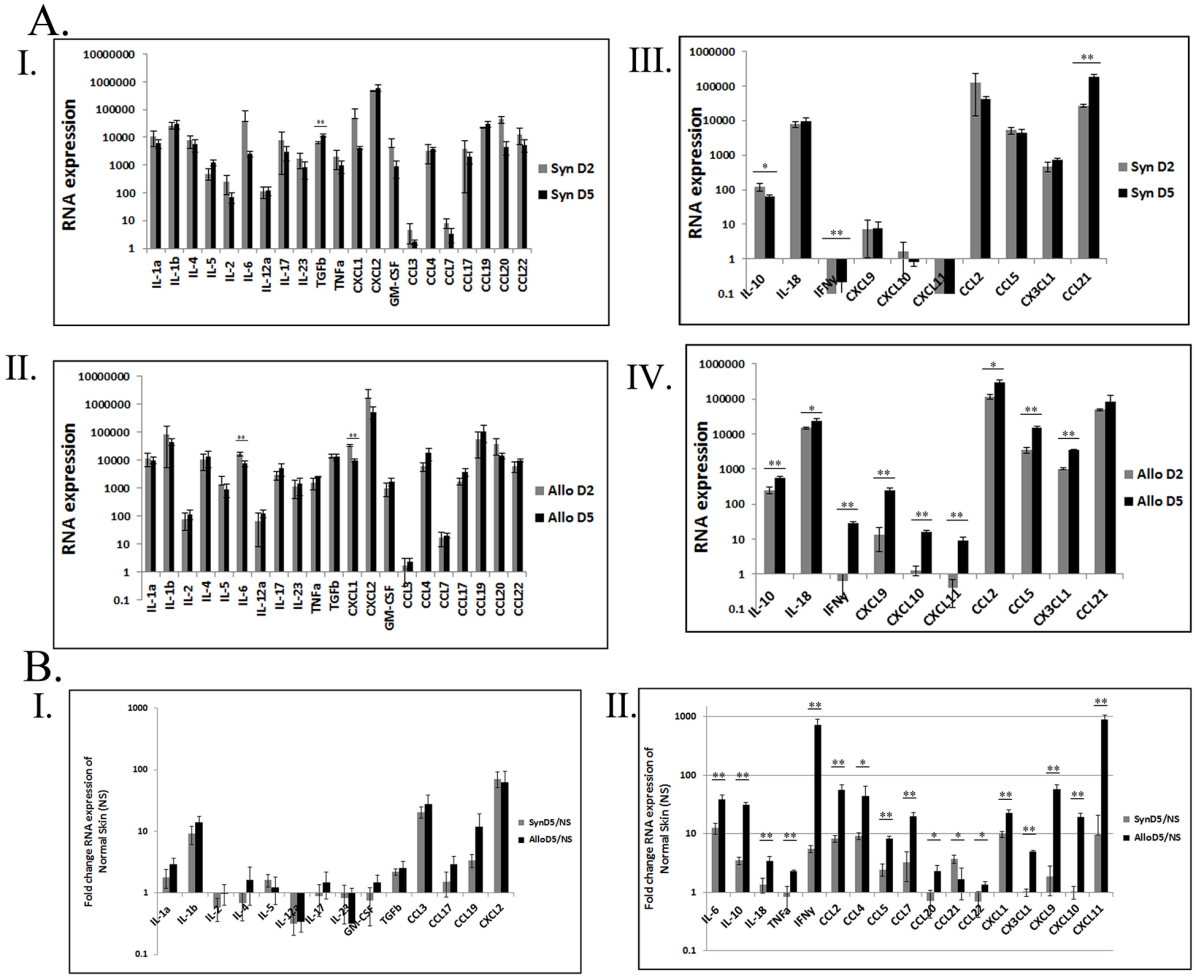
Comparison of the expression changes of an array of immune modulators between syngrafts and allograft from POD 2 to POD 5. Samples (n = 3 per group) from the graft-recipient intersection of syngrafts, allografts or NS were removed at on POD 2 and POD 5. RNA was extracted and gene expression analysis was performed by qRTPCR. (A) Comparison of gene expression levels on POD 2 versus POD 5, in both syngrafts and allografts. Genes displaying a similar gene expression pattern on POD 2 and POD5, in syngrafts (I) and allografts (II). Genes displaying a different gene expression pattern on POD 2 and POD 5, in syngrafts (III) and allografts (IV). (B) Comparison of the fold-changes in gene expression levels, with respect to NS, in syngrafts versus allografts. Genes displaying either nonsignificant (I) or a statistically significant (II) difference in expression between syngraft and allograft. * P<0/05 and ** P<0.01.

In order to confirm the grouping into injury-associated inflammatory versus anti-alloantigen response-related genes, we compared the changes in RNA expression levels following transplantation of both graft types. Most of the group 1 genes indeed displayed a similar expression pattern on POD 2 and POD 5 in both syngrafts (Fig 6AI) and allografts (Fig 6AII), confirming their involvement in injury-related inflammatory processes common to both graft types. Interestingly, the expression of both IL-6 and CXCL1 declined between POD 2 and POD 5 in both syngrafts and allografts, indicating a dedicated role in the early stages of the inflammatory response. In contrast, the expression of IFNg, CCL2, CCL5 and IL-10 from group 1 demonstrated a significant increase between POD 2 and POD 5 in the allograft only, indicating their involvement in anti-alloantigen response in addition to the damage-related response (Fig 6AIV). Similarly, between POD 2 and POD 5, most of the genes in group 2, IL-18, CXCL9, CXCL10, CXCL11 and CX3CL1 (Fractalkine), demonstrated a significant increase in allografts only (Fig 6AIV), echoed by enhanced recruitment of CD8^+^ and CD4^+^ cells, confirming their involvement in anti-alloantigen response processes (Figs 4 and 5). A summary of the final division of the different immune modulators into groups, considering both their expression relative to NS and their temporal changes, is displayed in Table 3. Interestingly POD 5 RNA levels of IL-6, IL-10, IL-18, IFNg, TNFa, CCL2, CCL4, CCL5, CCL7, CCL20, CCL22, CXCL1, CX3CL1, CXCL9, CXCL10 and CXCL11 were significantly higher in the allogeneic versus syngeneic grafts, (Fig 6BII) and agreed with the immunohistochemistry results (Figs 2–5), indicating a more robust immune response in the allograft. Initiation of the adaptive immune response was already evident at POD2 by the increased expression of IL-18, CX3CL1 and CXCL11 in the allograft compared to the syngraft (S3 Table) and in the infiltration of CD8^+^ cells to the allograft only (Figs 4 and 5).

**Table 3:** Grouping of the examined genes according to their expression in the syngeneic and allogeneic grafts.

1. Damage related inflammation	2. Anti-alloantigen reaction	3. Uninvolved genes
IL-1a*	**IL-18**	**IL-2**
**IL-1b**	**TNFa**	**IL-12**
**IL-6**	**CX3CL1**	**GM-CSF**
**TGFb**	**CXCL9**	**IL-17**
**CCL3**	**CXCL10**	**IL-23**
**CCL4**	**CXCL11**	**IL-4**
**CXCL1**	**CCL7**	**IL-5**
**CXCL2**		**CCL20**
**IL-10**	**IL-10** #	**CCL22**
**CCL2**	**CCL2** #	
**CCL5**	**CCL5** #	
**IFNg**	**IFNg** #	

^#^ Genes that demonstrated a statistically significant increase in their expression between POD 2 and POD 5 only in the allograft.

* Increased expression of the gene over NS was demonstrated in both grafts but was statistically significant only in the allograft.

### Protein expression profiles of syngrafts and allografts confirm distinct damage-related and anti-alloantigen responses during allograft rejection

**Fig 7:**
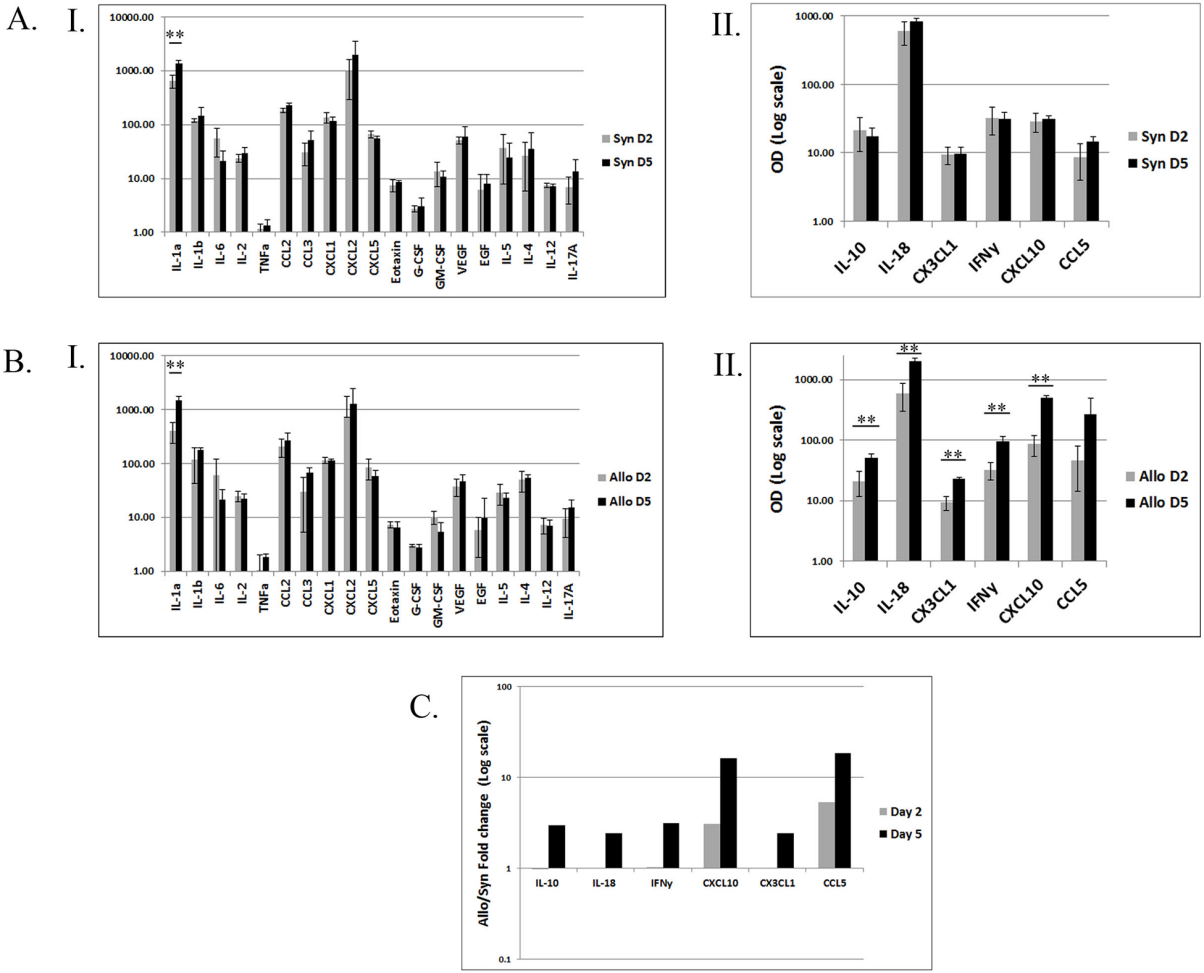
Protein analysis of syngrafts and allografts confirms the division of immune modulators into damage related and anti-alloantigen related groups suggested by the RNA analysis. Samples (n = 3 per group) from the graft-recipient interface of syngrafts and allografts were removed on POD 2 and POD 5, protein was extracted and a multiplex expression analysis was performed. Comparison of the POD 2 versus POD 5 gene expression levels in syngrafts (A) and allografts (B). Genes displaying a similar expression pattern on both days, in syngrafts (AI) and allografts (BI). Genes displaying a different gene expression pattern on both days, in syngrafts (AII) and allografts (BII). (C) Comparison of the fold changes in protein expression of syngraft and allograft between POD 2 and POD 5. * P<0/05 and ** P<0.01.

Next, proteins were extracted from syngeneic and allogeneic grafts and subjected to a multiplex assay of soluble factors (cytokines, chemokines and growth factors). Among the 25 examined factors, 19 factors, including cytokines (IL-1a, IL-1b, IL-2, IL-6, GM-CSF, TNFa, G-CSF, IL-4, IL-5, IL-12 and IL-17a), chemokines (CCL2, CCL3, CXCL1, CXCL-2 and CXCL5) and growth factors (EGF and VEGF), demonstrated almost identical patterns of expression in syngeneic and allogeneic grafts, on both POD 2 and POD 5 (Fig 7AI and 7BI). Importantly most of the factors displaying similar expression patterns in syngrafts and allografts belonged to group 1 (damage related inflammation) and group 3 (uninvolved genes), further supporting their roles suggested above (Table 3). Amongst these factors, IL-1a was the only factor that demonstrated a significant increase between POD 2 and POD 5, in both syngeneic and allogeneic grafts. In sharp contrast, and in line with the RNA expression profiles, six factors IL-18, IFNg, CXCL10, CCL5, CX3CL1 and IL-10, all from gene group 2 (anti-alloantigen response), were significantly upregulated between POD 2 and POD 5 in the allogeneic grafts, while their expression remained unchanged in the syngeneic grafts (Figs 6AIV and 7AII and 7BII). Similarly to the RNA data, when comparing the expression level of these factors between allogeneic and syngeneic grafts, we found that both CXCL10 and CCL5 already demonstrated increased expression on POD 2 (Fig 7C). This early increase in chemokine expression levels correlated with the increased infiltration of CD8^+^ cells into the allograft on POD 2 (Figs 4 and 5) and the initiation of the adaptive immunity response phase.

## Discussion

The current study aimed to identify factors specifically associated with the acute inflammatory versus anti-alloantigen components of the VCA rejection process, by comparing it to the inflammatory response provoked following implantation of a vascularized syngeneic graft involving an identical degree of injury. The initiation of immune cell infiltration on POD 2 was demonstrated at the graft-recipient border (the damage site) in the allograft that highly resembled the acute inflammatory response observed at the syngeneic graft which might suggest a possible role of injury-related inflammation in the induction of allograft rejection processes. Comparison of the expression pattern of a large array of immune modulators between allografts and syngrafts, identified a group of agents which seemingly play a common role in controlling the tissue injury-induced inflammatory responses in both graft types. At the same time, distinct RNA and protein expression profiles unique to the allografts, were noted and were ascribed to the anti-alloantigen graft rejection adaptive immune response. More specifically, IL-18, IFNg, CXCL9, 10 and 11, CCL2, CCL5, CX3CL1 and IL-10 were found to be upregulated in the allograft only. These profiles correlated with the progression of allograft rejection and their gene products were therefore suggested to be the main modulators of the anti-alloantigen response. Importantly, these factors have also been implicated in solid organ allograft rejection [28–35]. The development of a full-scale anti-alloantigenic response was apparent from the infiltration of immune cells into the entire allograft at POD 5, while the cell infiltrate in POD 5 syngrafts remained confined to the graft-recipient, as typically observed in wound healing responses.

### A possible role for tissue damage-induced inflammation in VCA rejection

Tissue damage-induced inflammation is a core event in early wound healing responses, and is triggered by the release of various DAMPs [18]. The role of damage response in allograft rejection has recently been demonstrated [19–21]. IRI-injured tissue serves as the primary source of DAMPs during whole internal organ transplantations (i.e., kidney, heart, liver), although limited surgery-induced damage also exists [26, 36, 37]. Augmented inflammation in organs from brain dead donors has been associated with both DGF and higher rates of rejection in kidney transplant patients [23]. Unlike internal organ transplantations, VCAs evoke extensive surgical damage at both the grafting site and at the outer perimeter of the graft, in the recipient and donor, respectively. In keeping with these reports, we observed initiation of inflammation, manifested by the infiltration of immune cells, at the damaged donor-recipient border. This damage-induced inflammatory response following allograft transplantation, involved granulocytes, macrophages, and CD4^+^ and CD8^+^ cells on POD 2, intensified by POD 5 and spread to the majority of the graft, providing fertile ground the initiation of the adaptive immune response.

**Fig 8:**
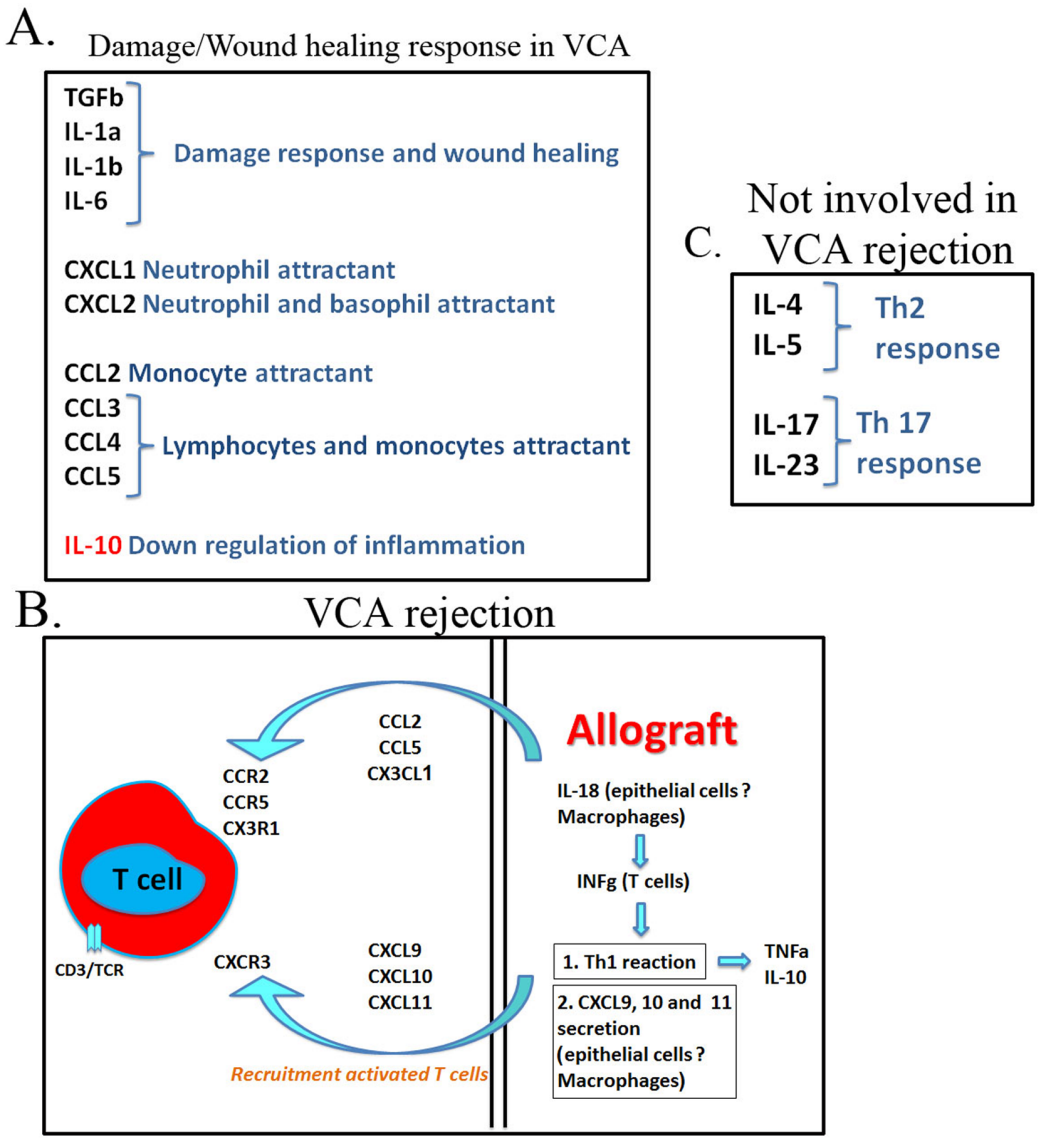
Schematic summary of the genes that control the wound healing and the anti-alloantigen components of VCA rejection. (A) Genes upregulated in both syngraft and allografts suggested to participate in the wound healing response following transplantation. (B) A schematic representation of VCA rejection process, as determined by the genes specifically upregulated during allograft rejection in the current study and in solid allograft rejection in earlier studies. (C) Th2 and Th17 phenotypes were not upregulated compared to NS and seemingly play no role in VCA rejection.

Damage-associated inflammation is primarily governed by cytokines that control the swift recruitment of granulocytes and later of macrophages and lymphocytes [16, 18]. The initial peak of these immune modulators typically appears within 24 hours of injury and disappears from the wound site within a few days [38, 39]. The expression patterns of TGFb, IL-1a, IL-6 and CXCL1, observed following VCA transplantation in our model followed the initial peak pattern and matched those reported during normal progression of wound healing inflammation [39–41]. In contrast high levels of many immune modulators can be detected for longer periods following injury, often mirroring the continuous infiltration of macrophages and lymphocytes [39–46]. In accordance, pronounced IL-1b, CCL2, CCL3, CCL4, CCL5 and CXCL2 expression was observed in both syngrafts and allografts throughout the monitoring period (Table 1 and S2 Table). Importantly, however, some of the evaluated immune modulators displayed significantly higher expression levels in POD 5 allografts, as compared to POD 5 syngrafts (TNFa, IL-6, CXCL1, CCL2, CCL4 and CCL5), that correlated with the dramatic progression of allograft rejection at this stage and was likely influenced by the anti-alloantigen-specific response at this stage. Expression profiles of the major cytokines and chemokines in allograft transplantation that were in agreement with normal damage-related inflammation are summarized in Fig 8A [39–46].

### High resemblance between specific anti-alloantigen responses that controls VCA and solid-organ rejections

Comparison of syngraft and allograft post-transplantation responses enabled identification of a group of immune modulators that were specifically upregulated in the allograft only (i.e., IL-18, IFNg, TNFa, CXCL9, 10 and 11, CCL2, CCL5, CX3CL1 and IL-10). Importantly, most of these factors, including IFNg [32], CXCL9, 10 and 11 [29, 30, 47], CCL2 and CCL5 [31, 34], were previously shown to be major players in T cell recruitment and consequently, in allograft rejection after solid organ transplantation. In an elegant set of experiments using various knockout mouse strains, Hancock demonstrated that the most important axes in implanted heart rejection were the chemokine receptors CXCR3, binding CXCL9, 10 and 11, and CCR5, binding CCL3, 4 and 5, which mediate recruitment of activated Th1 cells to the allograft [28]. This is in agreement with the specific upregulation of CXCL9, 10, 11, CCL2, and CCL5 in allografts in the current study. Increased production of IL-18 by macrophages or endothelial cells was demonstrated during both solid allograft [35, 48] and VCA rejection [49] and is known to induce IFNg expression and to promote Th1 differentiation [50]. CX3CL1 is expressed both in soluble and membranous forms by endothelial cells, enhancing capture of CX3CR1-expressing cells (i.e., CD8 cytotoxic and natural killer (NK) cells) in inflamed tissue and inducing their IFNg expression [51]. Enhanced expression of CXC3CL1 in cardiac allografts was previously observed in a mouse model [33] and was suggested as a urinary marker for human kidney rejection [52]. The deletion of the CXC3CL1 receptor in a mouse model, in combination with cyclosporin treatment, prolonged allograft survival [53]. The main function of IL-10 is in downregulation of the immune response. Recent reports have demonstrated the role of IL-10-expressing Th1 cells in inhibition of damage by exaggerated inflammation [54, 55]. Given the predominant Th1 phenotype in the VCA rejection observed in the current study, we suggest that the high IL-10 levels originated from Th1 cells. Importantly, a similar increase in IL-10 expression was observed during kidney rejection [56].

Taken together, VCA rejection closely resembles solid organ rejection, with a predominant Th1 phenotype (summarized in Fig 8B) governed mainly by high IFNg expression within the allograft, possibly induced by IL-18 upregulation that consequently induces CXCL9, 10 and 11 expression and the recruitment of activated T cells (primarily Th1 cells). Recruitment and capture of activated T cells (Th1) and NK cells is further augmented by CCL5 and CX3CL1 expression within the allograft. The seeming predominance of a Th1 immune response is further supported by the fact that no significant increase in expression of Th2-related cytokines (IL-4 and IL-5) or of Th17 cytokines (IL-17 and IL-23) was observed (Fig 8C).

## Conclusions

Accumulating data suggest that injury-induced inflammation plays a pivotal role in the initiation and progression of allograft rejection [19, 24]. The injury associated response that is initiated by this damage may speed up the recruitment of immune cells to the donor tissue and intensify allograft rejection. Our findings indicate to a possible involvement of damage induced inflammation in VCA rejection. Thus we speculate that the high rejection rates following VCA may arise, at least in part, from the pronounced damage response they provoke compared to solid organs. Future research focusing on the impact of agents (i.e., DAMPs receptors inhibitors) that inhibit damage-induced responses during and following VCA, may assist in reducing the occurrence of acute rejection episodes within the clinical settings. Our wide scope characterization of the major immune modulators governing VCA rejection may assist in defining specific markers for early detection of acute rejection episodes.

## Supporting information

S1 Table Primers used for qRT-PCR. (DOCX)

S2 Table Comparison of the RNA expression of an array of immune modulators of syngeneic or allogeneic grafts 2 days post transplantation with normal (uninflamed) skin (NS). (DOCX)

S3 Table Comparison of the fold-changes in gene expression levels at POD 2, with respect to NS, in syngrafts versus allografts. (DOCX)

S1 Fig POD 2 samples collected from the center of the allograft display no skin inflammation. Paraffin sections of POD 2 samples from the middle of the transplanted allografts were stained with H&E. A panoramic view made by stitching images, is presented. Insets of the original images are displayed to provide a higher image resolution of selected regions. No significant inflammation was detected.
(TIF)

S1 File NC3Rs ARRIVE guidelines checklist. (PDF)

## Abbreviations

BN: Brown Norway
DAMPs: Damage-associated molecular patterns
DGF: Delayed graft function
IRI: Ischemia-reperfusion injury
NS: Normal (uninflamed) skin
NLRs: Nucleotide-binding oligomerization domain-like receptors
POD: Post-operative day
RAGE: Receptor for advanced glycation end products
TLRs: Toll-like receptors
VCA: Vascularized composite allotransplantation
