# Supplementary material for: Immunological and inflammatory mapping of vascularized composite allograft rejection processes in a rat model
Source: PLoS One. 2017 Jul 26;12(7):e0181507. doi: 10.1371/journal.pone.0181507 (PMC5528841; doi:10.1371/journal.pone.0181507)
Supplement: S1 Table — (DOCX) [file pone.0181507.s001.docx]

| **Gene** | **Forward primers** | **Reverse primer** |
| --- | --- | --- |
| IL-1α | GAGATTCCGGAAACACCAAA | GAAAGCTGCGGATGTGAAGT |
| IL-1β | CAGGAAGGCAGTGTCACTCA | AAAGAAGGTGCTTGGGTCCT |
| IL-2 | AAGGAAACACAGCAGCACCT | GTGAGCATCATGGGGAGTTT |
| IL-4 | AGGGTGCTTCGCAAATTTTA | GTGAGTTCAGACCGCTGACA |
| IL-5 | CGATGAGGCTTCCTGTTCCT | CCCCCTCGGACAGTTTGATT |
| IL-6 | ACCACCCACAACAGACCAGT | CAGAATTGCCATTGCACAAC |
| IL-10 | CATCCGGGGTGACAATAACT | TGTCCAGCTGGTCCTTCTTT |
| IL-12β | CCTGAAGTGTGAAGCACCAA | GAGACTCAGGGGAACTGCTG |
| IL-12α | GCCTGCTTACCACTGGAACT | GCAGGCAGCTCCCTCTTATT |
| IL-17α | GCAAGAGATCCTGGTCCTGA | TGGCGGACAATAGAGGAAAC |
| IL-18 | ACCGCAGTAATACGGAGCAT | CAGTCTGGTCTGGGATTCGT |
| IL-23α | CAGCGTTCTCTTCTCCGTTC | GGCACTAAGGGCTCAGTCAG |
| GM-CSF | GACCCGCCTGAAGCTATACA | GAGGGCAGTTCGTCTGGTAG |
| TGF-β | GGACTCTCCACCTGCAAGAC | GACTGGCGAGCCTTAGTTTG |
| TNFα | GAGGTCAACCTGCCCAAGTA | GCTGGGTAGAGAACGGATGA |
| IFNγ | GCCCTCTCTGGCTGTTACTG | CCAAGAGGAGGCTCTTTCCT |
| CCL2 | GATGCAGTTAATGCCCCACT | ACCTGCTGCTGGTGATTCTC |
| CCL3 | CTTCTCCTATGGACGGCAAA | CGGTTTCTCTTGGTCAGGAA |
| CCL4 | CACCTCCCGGAAGATTCAT | CACAGATTTGCCTGCCTTT |
| CCL5 | ATATGGCTCGGACACCACTC | ACTGCAAGGTTGGAGCACTT |
| CCL7 | CTCCAAAGCCCTGAAGACAG | ACCGTAGTCCACCCATTTCA |
| CCL17 | CAATGTAGGCCGAGAGTGCT | CATCCTTGGGACACTCCACT |
| CCL19 | CAGGAAACCAAGGACCAGAA | TGCTCACACTCACGTTCACA |
| CCL20 | AGTCGAAAAGATCCGTGTGC | TTCCATCCCAGAAAAGCATC |
| CCL21 | ACTGCAGGAAGAATCGAGGA | TCACTGGGATACCCTCTTGG |
| CCL22 | GGATGCTCTGGGTGAAGAAG | TGGCAGAAGGATAGGGTTTG |
| NOS2 | GCTACACTTCCAACGCAACA | CATGGTGAACACGTTCTTGG |
| CXCL2 | TGGTTCAGAGGATCGTCCA | TCTTTGATTCTGCCCGTTG |
| CXCL1 | AAGGTGTGGGCACTGAAGTC | GCTCCCTCCTTGATGGTGTA |
| CXCL9 | AAGCAAAAGAGGGGGAAAAA | GCGCTTGTTGGTAAAGTGGT |
| CXCL10 | TGTCCGCATGTTGAGATCAT | GGCTCACCGCTTTCAATAAG |
| CXCL11 | CTCGCCTCATAATGCAGACA | CAAGACAGGAGAGGGTCAGC |
| CX3CL1 | ATCTGTGTACTCTGCTGGCG | AGCAAGGTCACTGGGATTGG |
| HPRT1 | TTGGTCAAGCAGTACAGCCC | TGGCCTGTATCCAACACTTCG |
| Rn18s | CGAAAGCATTTGCCAAGAAT | AGTCGGCATCGTTTATGGTC |

**S1Table.** **Primers used for qRT-PCR**.
