# Supplementary material for: Immunological and inflammatory mapping of vascularized composite allograft rejection processes in a rat model
Source: PLoS One. 2017 Jul 26;12(7):e0181507. doi: 10.1371/journal.pone.0181507 (PMC5528841; doi:10.1371/journal.pone.0181507)
Supplement: S2 Table — (DOCX) [file pone.0181507.s002.docx]

|  | **Average NS** | **Average Syn D2** | **Fold Av Syn D2/Av NS** | **P** |  | **Average NS** | **Average Allo D2** | **Fold Av Allo D2/Av NS** | **P** |
| --- | --- | --- | --- | --- | --- | --- | --- | --- | --- |
| **IL-1a** | **3395.48** | **10458.13** | **3.08** | **0.2195** | **IL-1a** | **3395.48** | **11857.17** | **3.49** | **0.0770** |
| **IL-1b** | **3220.67** | **26517.68** | **8.23** | **0.1321** | **IL-1b** | **3220.67** | **84898.17** | **26.36** | **0.1502** |
| **IL-2** | **119.86** | **256.20** | **2.14** | **0.3794** | **IL-2** | **119.86** | **79.23** | **0.66** | **0.4954** |
| **IL-4** | **8213.45** | **10363.59** | **2.14** | **0.5898** | **IL-4** | **8213.45** | **7744.66** | **0.94** | **0.8596** |
| **IL-5** | **750.94** | **496.14** | **1.26** | **0.2228** | **IL-5** | **750.94** | **1295.36** | **1.72** | **0.6128** |
| **IL-6** | **197.84** | **36140.06** | **182.67** | **0.3021** | **IL-6** | **197.84** | **16693.65** | **84.38** | **0.0002** |
| **IL-10** | **17.94** | **124.22** | **6.92** | **0.0240** | **IL-10** | **17.94** | **252.13** | **14.05** | **0.0084** |
| **IL-12a** | **375.13** | **112.47** | **0.30** | **0.0049** | **IL-12a** | **375.13** | **68.02** | **0.18** | **0.0035** |
| **IL-18** | **6951.90** | **7738.63** | **1.11** | **0.8212** | **IL-18** | **6951.90** | **14627.03** | **2.10** | **0.0748** |
| **IL-17** | **3460.22** | **7986.84** | **2.31** | **0.3736** | **IL-17** | **3460.22** | **2908.53** | **0.84** | **0.7038** |
| **IL-23** | **1000.43** | **1718.66** | **1.72** | **0.2622** | **IL-23** | **1000.43** | **1154.62** | **1.15** | **0.7390** |
| **TNFa** | **1126.60** | **2057.44** | **1.83** | **0.4054** | **TNFa** | **1126.60** | **1551.45** | **1.38** | **0.4650** |
| **IFNy** | **188.84** | **225.78** | **1.20** | **0.7574** | **IFNy** | **188.84** | **3045.08** | **16.12** | **0.1131** |
| **GM-CSF** | **1194.25** | **4139.72** | **3.47** | **0.3742** | **GM-CSF** | **1194.25** | **769.77** | **0.64** | **0.5388** |
| **TGFb** | **5188.24** | **6336.66** | **1.22** | **0.5546** | **TGFb** | **5188.24** | **14002.16** | **2.70** | **0.0172** |
| **CCL2** | **5187.19** | **122208.76** | **23.56** | **0.1078** | **CCL2** | **5187.19** | **118287.55** | **22.80** | **0.0005** |
| **CCL3** | **0.08** | **3.03** | **36.42** | **0.2008** | **CCL3** | **0.08** | **1.72** | **20.73** | **0.1148** |
| **CCL4** | **404.64** | **3284.83** | **8.12** | **0.0875** | **CCL4** | **404.64** | **5994.47** | **14.81** | **0.0084** |
| **CCL5** | **1842.43** | **5274.55** | **2.86** | **0.1488** | **CCL5** | **1842.43** | **3404.71** | **1.85** | **0.0332** |
| **CCL7** | **1.03** | **8.33** | **8.07** | **0.0152** | **CCL7** | **1.03** | **17.91** | **17.35** | **0.0412** |
| **CCL17** | **1290.01** | **3742.29** | **2.90** | **0.3190** | **CCL17** | **1290.01** | **1769.20** | **1.37** | **0.4287** |
| **CCL19** | **9177.97** | **22963.21** | **2.50** | **0.1932** | **CCL19** | **9177.97** | **56518.85** | **6.16** | **0.1470** |
| **CCL20** | **6314.21** | **44818.46** | **7.10** | **0.2183** | **CCL20** | **6314.21** | **38760.17** | **6.14** | **0.0605** |
| **CCL21** | **50131.12** | **26849.53** | **0.54** | **0.3577** | **CCL21** | **50131.12** | **50067.26** | **1.00** | **0.9979** |
| **CCL22** | **7567.91** | **13090.28** | **1.73** | **0.4533** | **CCL22** | **7567.91** | **6334.56** | **0.84** | **0.7324** |
| **CXCL1** | **426.28** | **37618.50** | **88.25** | **0.2165** | **CXCL1** | **426.28** | **33052.34** | **77.54** | **0.0000** |
| **CXCL2** | **8419.71** | **471296.50** | **55.98** | **0.1198** | **CXCL2** | **8419.71** | **1578510.65** | **187.48** | **0.2167** |
| **CX3CL1** | **717.31** | **477.09** | **0.67** | **0.3948** | **CX3CL1** | **717.31** | **1014.87** | **1.41** | **0.2085** |
| **CXCL9** | **4.16** | **7.02** | **1.69** | **0.4872** | **CXCL9** | **4.16** | **13.18** | **3.17** | **0.1608** |
| **CXCL10** | **0.81** | **1.68** | **2.08** | **0.3497** | **CXCL10** | **0.81** | **1.28** | **1.58** | **0.1429** |
| **CXCL11** | **0.01** | **0.02** | **2.26** | **0.2875** | **CXCL11** | **0.01** | **0.41** | **40.13** | **0.0809** |

**S2 Table. Comparison of the RNA expression of an array of immune modulators of syngeneic or allogeneic grafts 2 days post transplantation with normal (uninflamed) skin (NS).**
