# Supplementary material for: Immunological and inflammatory mapping of vascularized composite allograft rejection processes in a rat model
Source: PLoS One. 2017 Jul 26;12(7):e0181507. doi: 10.1371/journal.pone.0181507 (PMC5528841; doi:10.1371/journal.pone.0181507)
Supplement: S3 Table — (DOCX) [file pone.0181507.s003.docx]

|  | **Fold Av Syn2/Av NS** | **Fold Av Allo2/Av NS** | **P** |
| --- | --- | --- | --- |
| **IL-1a** | **3.08** | **3.49** | **0.82** |
| **IL-1b** | **8.23** | **26.36** | **0.29** |
| **IL-2** | **2.14** | **0.66** | **0.25** |
| **IL-6** | **182.67** | **84.38** | **0.56** |
| **IL-10** | **6.92** | **14.05** | **0.021** |
| **IL-17** | **2.31** | **0.84** | **0.31** |
| **IL-18** | **1.11** | **2.10** | **0.004** |
| **TNFa** | **1.83** | **1.38** | **0.65** |
| **IFNy** | **1.20** | **16.12** | **0.11** |
| **GM-CSF** | **3.47** | **0.64** | **0.32** |
| **TGFb** | **1.22** | **2.70** | **0.007** |
| **CCL2** | **23.56** | **22.80** | **0.95** |
| **CCL3** | **36.42** | **20.73** | **0.57** |
| **CCL4** | **8.12** | **14.81** | **0.19** |
| **CCL5** | **2.86** | **1.85** | **0.39** |
| **CCL7** | **8.07** | **17.35** | **0.18** |
| **CCL17** | **2.90** | **1.37** | **0.41** |
| **CCL19** | **2.50** | **6.16** | **0.27** |
| **CCL20** | **7.10** | **6.14** | **0.84** |
| **CCL21** | **0.54** | **1.00** | **0.002** |
| **CCL22** | **1.73** | **0.84** | **0.33** |
| **CXCL1** | **88.25** | **77.54** | **0.87** |
| **CXCL2** | **55.98** | **187.48** | **0.37** |
| **CX3CL1** | **0.67** | **1.41** | **0.033** |
| **CXCL9** | **1.69** | **3.17** | **0.37** |
| **CXCL10** | **2.08** | **1.58** | **0.66** |
| **CXCL11** | **2.26** | **40.13** | **0.09** |

**S3 Table. Comparison of the fold-changes in gene expression levels at POD 2, with respect to NS, in syngrafts versus allografts.**
