# Supplementary figures and images for: Immunological and inflammatory mapping of vascularized composite allograft rejection processes in a rat model
Source: PLoS One. 2017 Jul 26;12(7):e0181507. doi: 10.1371/journal.pone.0181507 (PMC5528841; doi:10.1371/journal.pone.0181507)

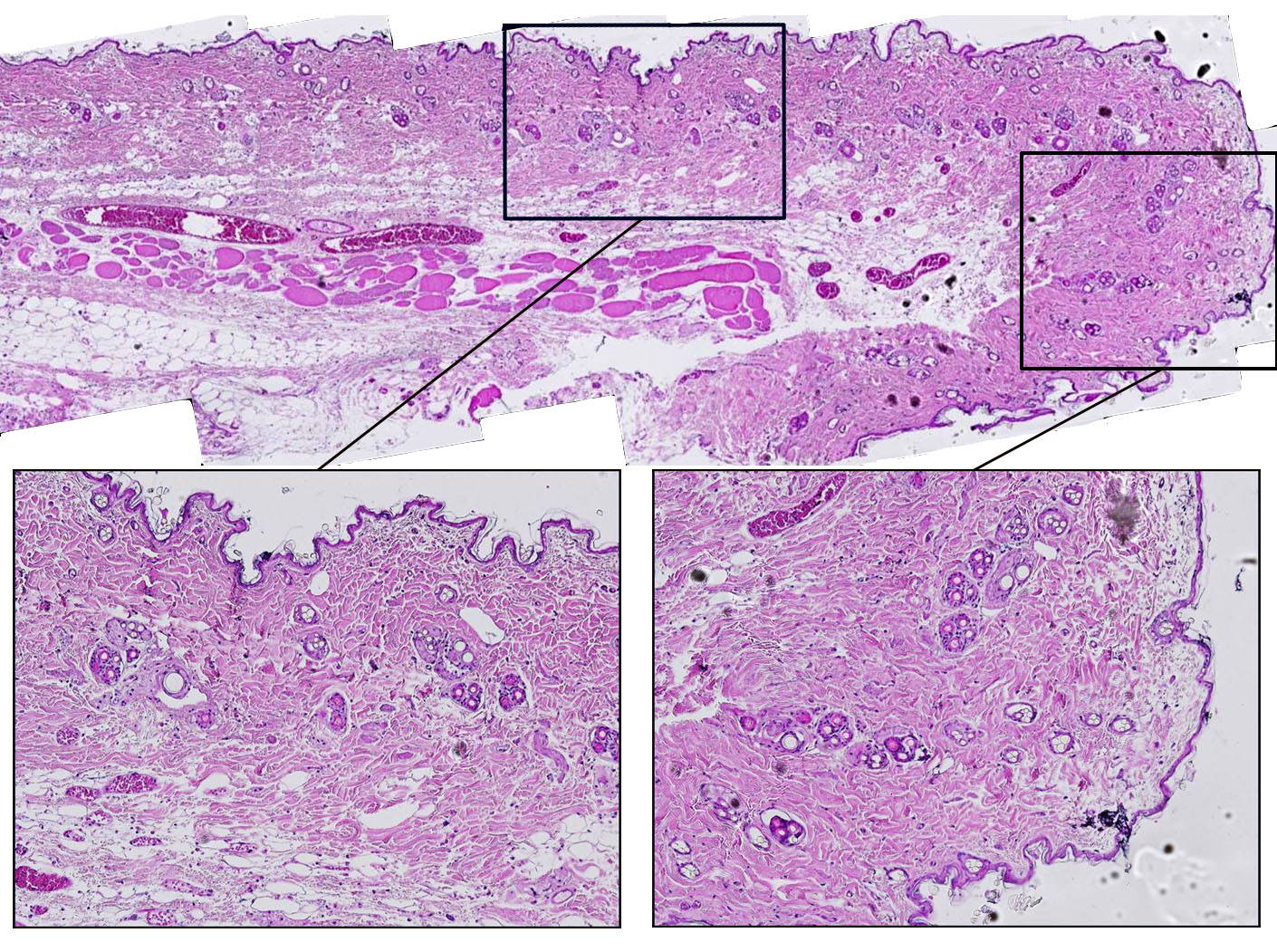

Supplement: S1 Fig — Paraffin sections of POD 2 samples from the middle of the transplanted allografts were stained with H&E. A panoramic view made by stitching images, is presented. Insets of the original images are displayed to provide a higher image resolution of selected regions. No significant inflammation was detected. (TIF) [file pone.0181507.s004.tif]
